# Tribocatalytically-active nickel/cobalt phosphorous films for universal protection in a hydrocarbon-rich environment

**DOI:** 10.1038/s41598-023-37531-0

**Published:** 2023-07-05

**Authors:** Asghar Shirani, Rawan Al Sulaimi, Ali Zayaan Macknojia, Mohammad Eskandari, Diana Berman

**Affiliations:** Department of Materials Science and Engineering, University of North Texas, Denton, TX 76203 USA

**Keywords:** Synthesis and processing, Structural properties, Materials science

## Abstract

High-contact stresses generated at the sliding interfaces during their relative movement provide a unique combination of local heating and shear- and load-induced compression conditions. These conditions, when involving the sliding of surfaces with certain material characteristics, may facilitate tribochemical reactions with the environment, leading to the formation of a protective, damage-suppressing tribofilm directly at the contact. Here, we employ the electrodeposition process to design a coating composed of a hard cobalt-phosphorous matrix with the inclusion of tribocatalytically-active nickel clusters. The coating is optimized in terms of its relative composition and mechanical characteristics. We demonstrate the excellent tribological performance of the coating in the presence of a hydrocarbon environment, both in the form of a liquid lubricant and as a hydrocarbon-saturated vapor. Characterization of the wear track indicates that the origin of such performance lies in the formation of a protective carbon-based tribofilm on the surface of the coating during sliding. These results contribute to the advancement of knowledge on material transformations in the contact, thus providing a robust and versatile approach to addressing tribological challenges in mechanical systems.

## Introduction

Surface degradation is believed to account for more than 70% of the causes for the loss of usefulness of mechanical systems^1,2^. Out of this, 50% of the causes are attributed to mechanically induced wear and deformation of materials^3,4^. To address this issue, surfaces are covered with protective films^5,6^. However, these, eventually wear out, and replenishment becomes a great challenge, as the deposition of coatings usually requires high temperature/high vacuum processing conditions that at unsuitable for large components in need of protection.

Local heating and shear- and load-induced compression of the sliding surfaces^7,8^, when involving a correct combination of materials, may induce tribochemical reactions^9^ that lead to the formation of a protective damage-suppressing tribofilm from the surrounding environment directly at the contact interface, sometimes referred to as tribocatalysis^8,10–14^. One of the most interesting and intriguing combinations of material systems experiencing such tribochemical activity involves carbon-based materials or composites in close proximity to catalytically active metals, such as copper, platinum, nickel, etc.^15,16^. In this case, the nature of the carbon source can be in gas, liquid, or solid form^15^.

The formation of carbon-based tribofilms is very attractive from a tribological perspective. Carbon-based materials and their combinations, such as, for example, polycrystalline diamond films, diamond-like carbon (DLC), or metal carbides, are already widely employed as protective coatings for the surfaces involved in sliding and rolling processes^17–19^. Prior studies also demonstrated that the lubricating carbon coatings can be represented in the form of a two-dimensional (2D) material, graphene^20–22^. Easy shear of 2D layers provides a unique set of characteristics needed for suppressing damage in mechanical contact^23–27^. Specifically, in the case of graphene platelets lubricating sliding steel counterparts, we observed a 4–5 times reduction in friction and 4 orders of magnitude reduction in wear^28,29^. Combining different forms of carbon, such as graphene and nanodiamonds, can even lead to a state of vanishing friction and wear, called superlubricity^30^. The automatic generation and replenishment of carbon-based films, therefore, can significantly improve the concept of surface protection.

While traditional approaches to the deposition of a carbon-based coating require elevated temperatures, dynamic processes that occur at the sliding interfaces create favorable high temperature and pressure conditions^7,31^ thus significantly reducing external temperature and energy supply needs. Once all the conditions are satisfied, the formation of very durable and self-generating boundary or surface films, usually in the form of DLC or graphitic films^15^, directly at the sliding contact can be observed^13,32^. For example, the formation of DLC films on platinum-gold surfaces was observed in a dry sliding environment with traces of organics^33^. Tribologically-induced transformation of the carbon-iron system during sliding in a dry environment, as shown in the case of iron nanoparticles dispersed on the silicon substrate surface during sliding against the DLC counterface, can facilitate the reconstruction of DLC amorphous carbon into onion-like carbon (OLC) structures^34^. Alternatively, DLC-like films were also generated by the incorporation of magnesium silicate hydroxide (MSH) nanoparticles, either in the form of additives in liquid lubricants^35^ or as burnished coatings^36^.

Tribocatalytic and tribochemical activities at the sliding interfaces became a very attractive topic for industrial application when the carbon source could be provided by the liquid lubricant^15,37–39^. In order to understand the role of the catalytic metal in the tribochemical activity, Erdemir et al.^38^ incorporated copper or nickel structures into the ceramic matrix. They stated that the presence of catalytically active metals in the composite coating subjected to sliding in oil initiates the growth of DLC film in the wear track^38^. This formed DLC significantly reduced the friction and wear of the sliding interfaces. Further, immersion of the Pt-containing^39^ and Cu-containing^40^ surfaces into alkanes during sliding resulted in the in-situ generation of the carbon-based tribofilms, with the structure and growth rate of the films being determined by the nature of the catalytic materials and the sliding load and temperature conditions. Specifically, it was shown that the observed tribofilm growth rate can be fitted to an Arrhenius equation, indicating an increase in the film formation with applied load and higher temperature^39^.

In this study, we focus on the development and improvement of a coating aimed at enhancing the tribological performance of mechanical systems operating in various hydrocarbon-rich environments. Specifically, we explore the tribochemical activity facilitated by catalytically-active materials, leading to the formation of protective tribofilms from hydrocarbon sources, and how this process enhances surface protection during sliding. Previous studies have indicated that tribofilm formation is influenced by the interactions between the coating and the environment, suggesting the need to adjust the coating composition based on the nature of the carbon precursor.

To address this issue, we utilize the electrodeposition process to design a novel type of tribocatalytically-active coating: a nickel–cobalt–phosphorous-based film, with nickel serving as the tribocatalysis activator and cobalt phosphorous acting as the supporting matrix. Our results demonstrate that the coating exhibits excellent tribological performance in both liquid and gaseous hydrocarbon-rich environments. We attribute the origin of these friction and wear-reducing characteristics to the tribocatalytically-driven formation of protective carbon films directly at the contact during sliding. Overall, our findings open up new possibilities for designing tribocatalytically-active coatings that exhibit universality across liquid and gaseous hydrocarbon-rich environments.

## Experimental procedure

### Deposition of the tribocatalytically active coatings

The coatings made of CoP matrix with the inclusion of active nickel elements were made on AISI 52100 steel and copper substrates using the electrodeposition process. Different relative compositions of the resulting coatings were analyzed to unravel the full potential of their tribocatalytic activity. The mirror-polished (~ 50 nm roughness) and hardened (~ 60 HRC) 52100 steel substrates were cleaned with acetone prior to the deposition. To remove any grease or residue that remained on the surface during storage, the deposition was preceded by anodic cleaning in a strong alkane solution. After cleaning, the substrate was submerged in 10% H_2_SO_4_ diluted acid at room temperature to improve the adhesion of the coating. The deposition was performed in an electrolyte composed of a mixture of CoCl_2_ (210 g/l), NiCl_2_ (20 g/l), H_3_PO_4_ (50 g/l), sodium hypophosphite (20 g/l), and sodium saccharin (1 g/l) using a Pt electrode at 2–3 V. The approximate 25 μm thickness of the resulting coating was controlled by the time of the deposition process***.***

### Tribological tests

Tribological tests were performed using an Anton Paar pin-on-disk TRB^3^ tribometer equipped with an enclosure for controlled environment tests. The uncoated 52100 steel and copper substrates purchased from McMaster Carr were used as baseline materials. To minimize the tribochemical activity at the counterface, all the tests were performed against alumina balls of 6 mm in diameter. The tests were performed at 50 °C in a reciprocating mode with a 1.4 mm stroke length at a 2 Hz frequency. The applied load and corresponding maximum contact pressure were in the range of 1–5 N (or maximum Hertzian pressure of 0.66–1.13 GPa). The tests were carried out using two different hydrocarbon-based sources: liquid lubricant in the form of decane and a gas environment in the form of an ethanol vapor/nitrogen mixture. In decane, the tests were performed by covering the samples with 150 cc of the lubricant. In the case of the ethanol vapor tests, the nitrogen gas was flowing through the ethanol bath in the home-built experimental setup to reach full saturation of the chamber enclosure with the ethanol vapor. The relative pressure ratio of ethanol vapor to nitrogen gas was ~ 1:17.

### Characterization

Chemical analysis and elemental mapping were performed using an FEI Quanta 200 SEM equipped with energy-dispersive X-ray spectroscopy (EDS). Analysis of the wear tracks and ball wear marks was performed using a Zeiss optical microscope. Coating roughness and thickness analysis were performed using a Veeco Dektak 150 stylus profilometer with a 2.5 μm tip radius. Raman characterization of the formed tribofilms was acquired using a Renishaw Raman Spectrometer equipped with a green laser (532 nm wavelength). 3D profilometry images were collected using the Filmetrics optical interferometer (KLA Instruments). Hardness analysis of the coatings has been performed using iNano nanoindenter (KLA Instruments).

## Results and discussion

**Figure 1:**
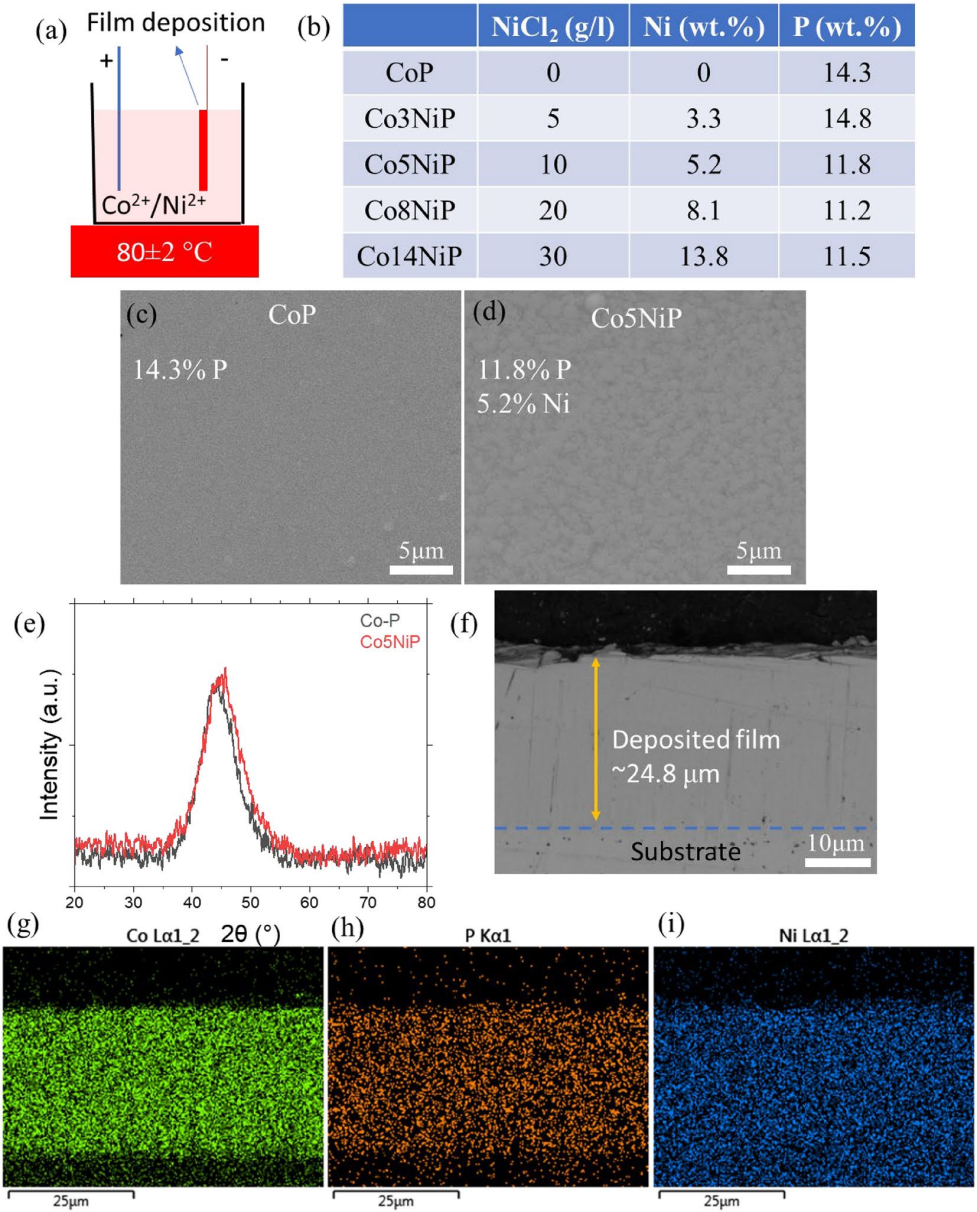
(**a**) Schematic of CoP, CoNiP electrodeposition. (**b**) summary of the resulting composition of the coatings. Surface texture (**c**) CoP and (**d**) Co5NiP. XRD of the deposited film (**e**). Micro-cross section of the CoP film shows the film's thickness (**f**) and the uniform distribution of (**g**) Co, (**h**) P, and (**i**) Ni within the film's thickness.

Mirror-like coatings (Fig. 1) with 5–20 wt.% Ni and Ra ~ 2 nm were co-electrodeposited by tuning the Co^+2^/Ni^2+^ ratio in the electrolyte through the setup schematically shown in Fig. 1a. In the absence of Ni ions, the CoP coating with 14 wt.% P was deposited, and after introducing Ni ions at the concentration of 5–25 g/l, a series of CoNiP coatings with 3.3 to 13.8 wt% of Ni were deposited on the substrates (Fig. 1b). The SEM images confirm the smooth appearance of the coatings without and with the addition of Ni (Fig. 1c, d). XRD analysis of the deposited coatings reveals the amorphous structure of the films in the case of pure CoP film and CoNiP alloys (Fig. 1e). The film thickness was measured to be 24.8 ± 0.5 µm (Fig. 1f), which is thick enough to reduce the effect of the substrate on the mechanical properties of the surfaces in contact. A uniform distribution of Ni and P across the film was detected in Fig. 1g–i.

**Figure 2:**
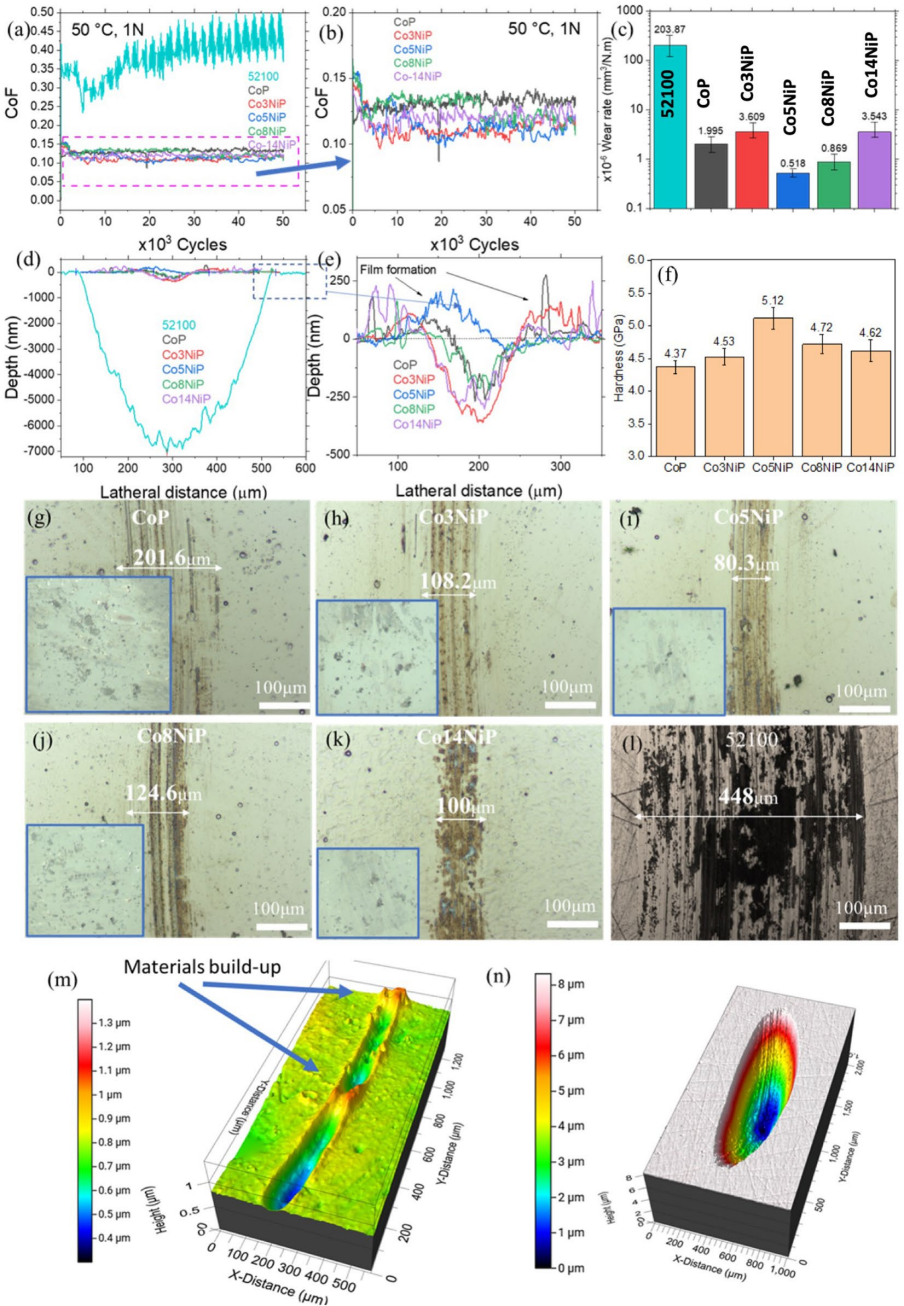
(**a,b**) Coefficient of friction of the CoP coatings with 3, 5, 8, and 14 wt% of nickel at 50 °C in contrast to the uncoated 52100 steel. due to the major difference between 52100 and CoNiP coatings’ CoF, the dashed area in (**a**) is replotted with different scales in (**b**). Significant improvements in both the COF and wear are demonstrated. (**c**) Calculated wear rate for the 52100 steel and coatings. (**d,e**) Stylus profilometry results for the wear track profiles captured at the center of the wear tracks. (**f**) Nanoindentation hardness of the coatings. (**g–l**) Optical micrographs of the wear tracks formed on CoP with 0, 3, 5, 8, and 14% Ni, and on the uncoated 52100 steel respectively. White light interferometer images of the (**m**) Co5NiP and (**n**) 52100 steel wear tracks showing the difference in the nature of the wear tracks.

To determine the optimum concentration of Ni in the coating, and get the most promising protection on the surface, the coatings were tested in a decane environment. Decane has been selected as an environment of interest since it presents one of the most promising low-viscosity fuels for next-generation high-efficiency combustion engines. The tribological experiments were conducted in decane at 1 N load and 50 °C temperature for 50k cycles (Fig. 2). Uncoated AISI 52100 steel was used as the reference point since this alloy is the most commonly used material in relevant industrial applications. The coefficient of friction (CoF) of the uncoated steel surface in a decane environment was measured to be 0.45; in the case of the coatings, the average CoF for CoNiP alloys was considerably lower, around 0.12 (Fig. 2a, b). The wear of the coatings changed depending on their composition (Fig. 2c).

The profilometry analysis of the wear tracks also indicates the substantial difference between the wear of the CoNiP coating and the uncoated steel substrate (Fig. 2d, e). The coating with the best performance also showed an improvement in mechanical characteristics (Fig. 2f). As confirmed by the profilometry results (Fig. 2d, e) and optical images (Fig. 2g–k) the whole CoNiP group demonstrated very similar wear track widths, with the lowest width observed for the Co5NiP (wear track width of 80.3 µm being close to the Hertz contact size). The steel substrate, on the other hand, has a wear track width of 448 µm (Fig. 2l). The best performance coating with 5% Ni has a wear rate of 2.18 × 10^–7^ mm^3^/N m, which is nearly three orders of magnitude lower than the steel with a wear rate of 2.038 × 10^–4^ mm^3^/N m.

In terms of the wear track depth, while the uncoated steel has a deep wear profile with a depth of ~ 7000 nm, all the members of the CoNiP group provide relatively shallow wear tracks with depths below 250 nm. Interestingly, variation in the CoF and wear behavior within the CoNiP group partially coincides with the mechanical characteristics of the coatings (Fig. 2f). Prior studies suggested that under identical testing conditions, samples that possess a lower friction coefficient and greater hardness exhibit reduced wear volumes^41^. Harder materials with lower adhesion tendencies and lower friction coefficients contribute to reduced wear volumes by minimizing contact area, material transfer, and wear debris formation. While the introduction of ~ 3 wt% of Ni (Co3NiP) led to a slight increase in wear relative to pure CoP film, the addition of 5 wt% of Ni (Co5NiP) enabled almost 4 times the wear reduction. A further increase in the nickel content decreased the hardness of the coating and increased its wear.

The intriguing observation during the profile analysis is finding areas with a film formed on top of the wear track, as indicated by the positive values in Fig. 2d. The minimum profile depth with the greatest tendency for film formation has been observed for Co5NiP film, which previously also indicated the lowest wear rate. This film formation is attributed to the tribocatalytic potential of the coating during sliding. As a result, the Co5NiP was selected for further analysis to determine the origin of such performance and the effect of the environment on the film formation.

**Figure 3:**
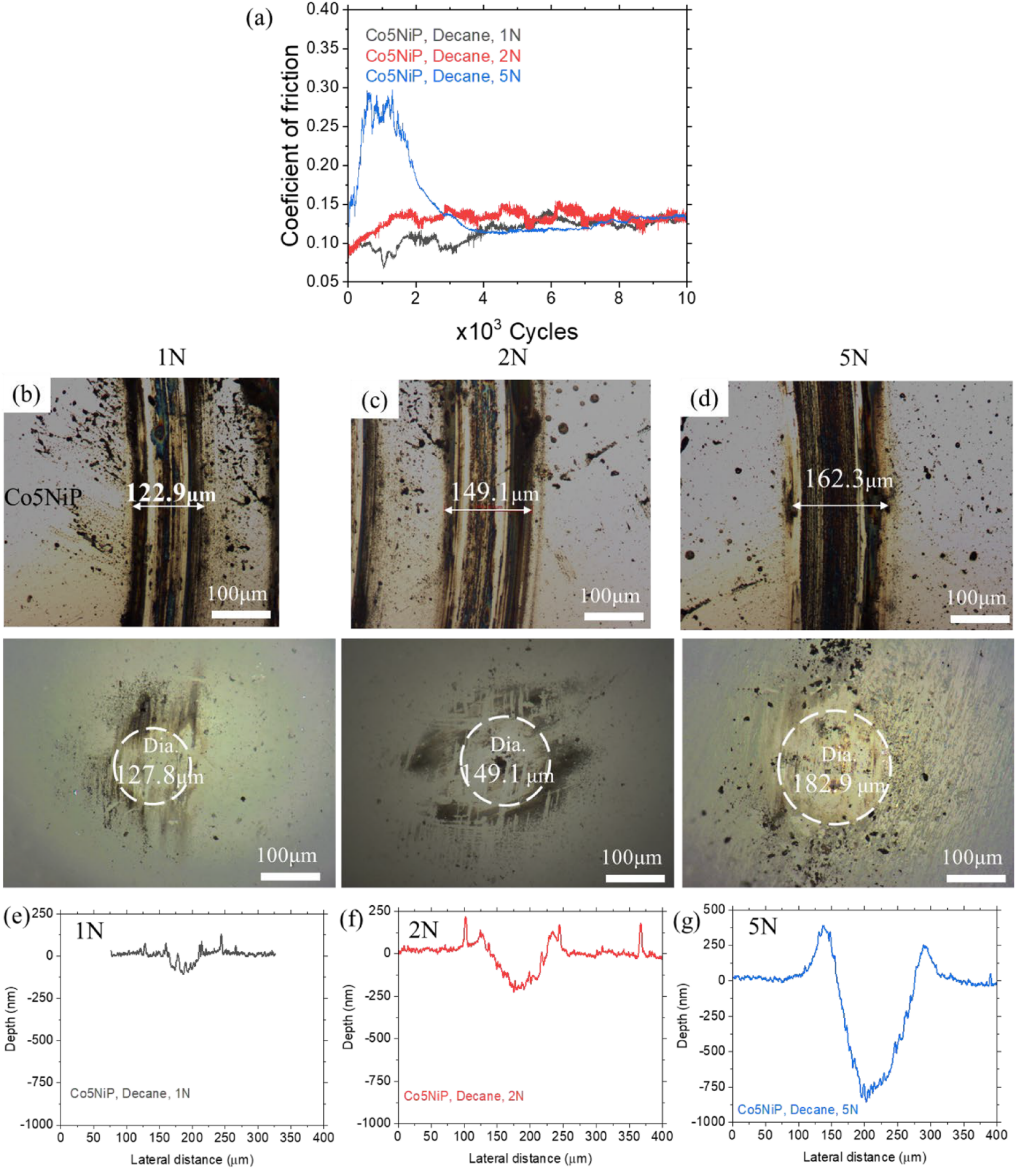
(**a**) Coefficient of friction of Co5NiP as a function of load upon sliding in *decane* at 25 °C*.* (**b–d**) Optical micrographs of the wear tracks and the corresponding counter-bodies after the testing in decane. (**e–g**) Profilometry analysis of the wear tracks formed during testing of the coated sample.

**Figure 4:**
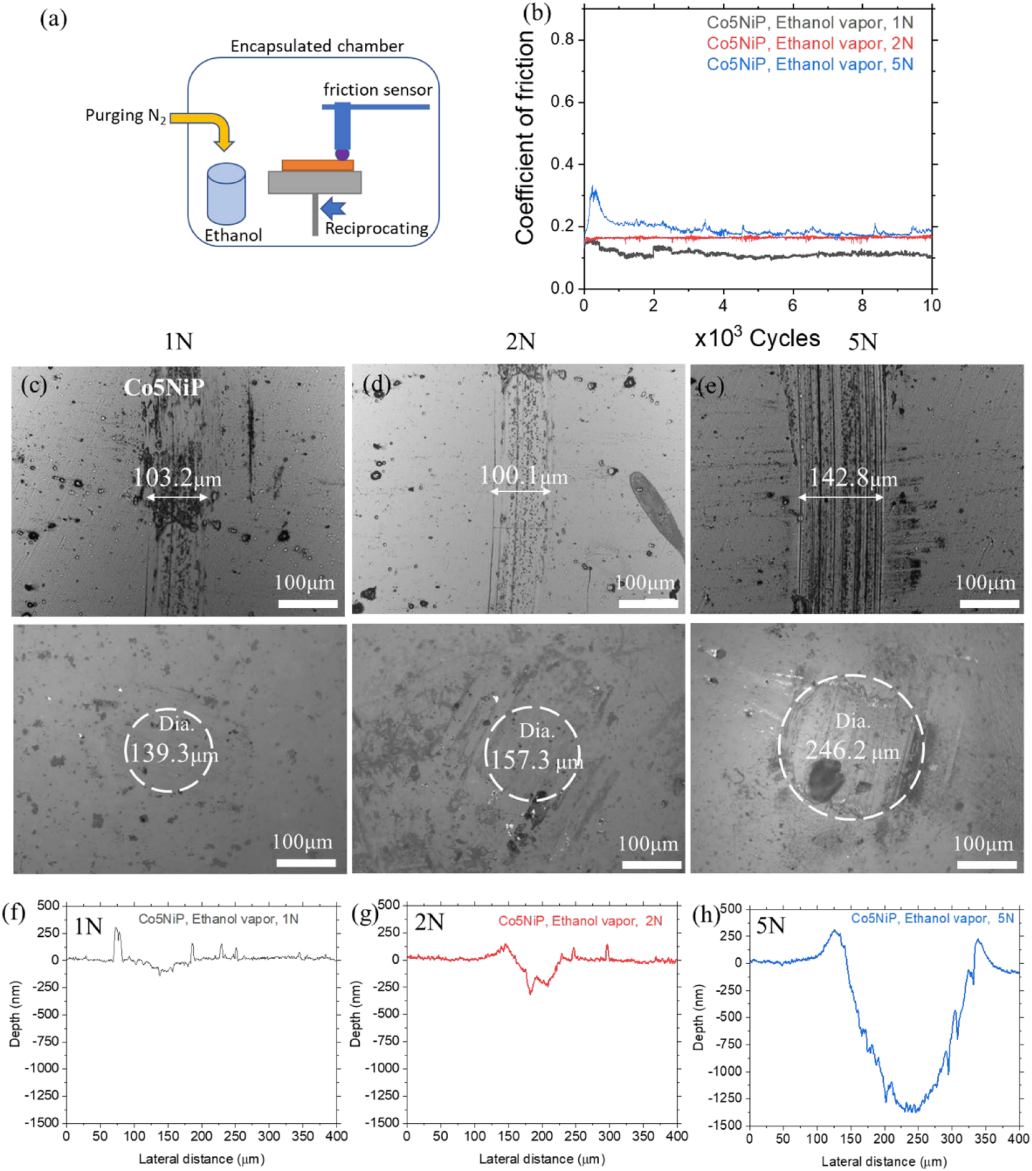
(**a**) Schematic of the tribology set-up used for N_2_/ethanol vapor tests at 25 °C. (**b**) Coefficient of friction for the Co5NiP coating as a function of load under exposure to ethanol vapor. (**c–e**) Optical micrographs of the wear tracks and counter-body after testing in ethanol vapor. (**f–h**) Profilometry analysis of the wear tracks formed during testing.

While the prior tests demonstrated improved tribological characteristics of the coatings at elevated temperature conditions, elevated temperatures are not always viable from an application standpoint. Therefore, we further tested the performance of the Co5NiP coating at room temperature to understand its potential to sustain wear and improve frictional behavior. The frictional behavior summaries of the selected Co5NiP coating in decane and ethanol vapor are illustrated in Figs. 3 and 4, respectively. Though in general the performance at room temperature slightly deteriorates relative to the elevated temperature conditions, the Co5NiP coating still shows relatively uniform and steady behavior as the load progresses within the 1–5 N range.

In decane, Co5NiP shows an average COF of 0.12 for a 1–5 N load (Fig. 3). Note that at 5N of load, the maximum contact pressure reaches 1.13 GPa which is considered to be high to generate larger wear. Therefore, we attribute the observed sudden increase in the CoF value to the generation of wear debris that act as a third body at the sliding interface. After producing debris, the self-protecting nature of the coating is activated through the release of catalytic clusters, thus facilitating the formation of the tribo-catalytic carbon film which leads to lowering the friction and prolonging the life span. At the same time, upon increasing the load from 1 to 5 N, the wear track width shows a slight enlargement from 122.9 to 162.3 µm, respectively (Fig. 3b–d). The cross-section profiles of the wear track are in agreement with these observations (Fig. 3e–g), demonstrating quite a low depth of the wear tracks falling within the coating thickness (up to 750 nm for 5 N of load). It should be noted that the uncoated 52100 steel at 1 N of load in decane, meanwhile, showed substantial nonuniformity of the worn surfaces around the edges of the wear track. The tribology results reveal good protection of the coated surface in decane media in contrast to the uncoated 52100 steel, even though the uncoated steel substrate has almost two times higher hardness than the coating.

To further unravel the universality of the Co5NiP coating for protecting surfaces in carbon-rich environments, the coating was tested in a gaseous media. To eliminate the effects of humidity and residual oxygen, the system was purged with N_2_ for 10 min before introducing ethanol vapor into the chamber. Prior to the experiment, to ensure a relatively uniform atmosphere within the chamber, the enclosure was filled with N_2_-ethanol vapor for at least 10 min. The tribology analysis of the wear tracks is presented in Fig. 4. A frictional study reveals that Co5NiP has a low CoF for the tests conducted at a 0.66–1.13 GPa maximum contact pressure range. Notably, the observed CoF values are similar for liquid and gaseous environments, as long as they are hydrocarbon-rich. Similarly to the decane experiments, increasing the load from 1 to 5 N increases the wear track width (from 103.2 to 142.8 µm). Analysis of the Co5NiP wear track cross-sections indicated signs of material accumulation, especially at the edges of the wear track. To validate the assumption that the origin of such tribofilms, both in decane and ethanol vapor, is connected to tribocatalysis, we performed further characterization of the wear tracks.

**Figure 5:**
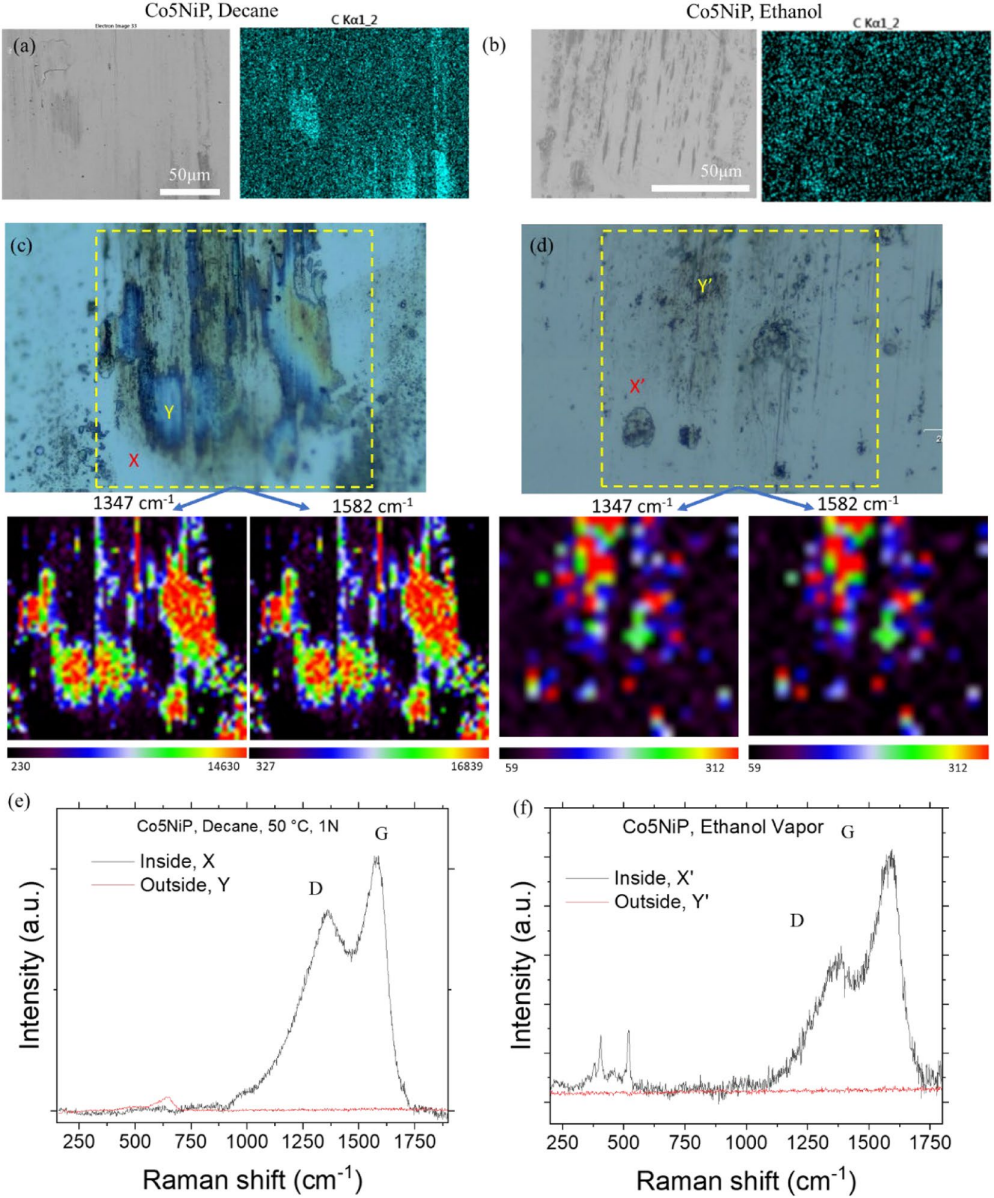
SEM and Raman analysis indicating the formation of carbon-rich tribofilms inside the wear track. (**a,b**) EDS mapping of the wear track tested in *decane* and ethanol vapor for Co5NiP coatings, respectively. (**c,d**) Optical micrographs showing the area for which the 2D-Raman spectroscopy was acquired for *decane* and ethanol vapor media, respectively. Single spectra of the selected points are noted with X, and Y for decane and ethanol vapor, respectively (**e, f**).

The micrographs (Fig. 5) of the wear track, along with the 2D elemental mapping (EDS) and Raman spectroscopy analysis, suggest that the origin of the beneficial tribological characteristics of the coating is indeed the in-situ formation of carbon-rich tribofilm. In both environments (decane and ethanol vapor), traces of strongly adhered carbon patches, with their concentration increasing toward the edges of the wear track, were detected. Raman spectroscopy indicated that these formed carbon layers have a mixture of D (at ~ 1350 cm^−1^) and G (at ~ 1560 cm^−1^) bands typical for the amorphous carbon structures or DLC structures, as described in previous studies^39–41^. A slight variation in the relative intensity of D and G peaks is attributed to the higher presence of not fully converted tribopolymers in a decane environment (as a result of decane molecule fragments) in contrast to ethanol. Interestingly, this variability in the nature of the tribofilm has a minimum effect on the effectiveness of the underlying surface protection, with the changes observed only at lower loads. The nature of the observed carbon films is similar to the ones reported in previous studies^41^.

The tribochemical reaction between the lubricant and the coating plays a dominant role in the formation mechanism of surface films, the resulting tribofilm contributes to the improved tribological properties of the coating^37^. The formation of the carbon tribofilm is crucial for minimizing friction and wear of the surfaces during sliding. This protective layer helps to distribute the applied load more evenly, reducing localized stress and preventing excessive material removal or wear. As the tribofilm gradually wears off, the nickel clusters within the coating become exposed to hydrocarbons once again, leading to the reactivation of tribocatalysis. Consequently, fresh layers of tribofilm form protecting the underlying surface from damage. This continuous and self-regulating process ensures ongoing protection and extended lifetime of the tribological surfaces.

## Conclusion

CoNiP coatings with 3–14 wt.% Ni incorporated in the CoP matrix were developed using the electrodeposition approach. Their tribological characteristics were tested to optimize the relative composition of the resulting materials, revealing that CoNiP with 5 wt.% Ni demonstrates optimal performance in a decane environment. The resulting coefficient of friction was reduced four times when compared to the uncoated 52100 steel surfaces. The wear analysis also showed that the Co5NiP coating demonstrated almost three orders of magnitude reduction in wear rate, from 5.18 × 10^–7^ mm^3^/N m for the coating in comparison to 2.038 × 10^–4^ mm^3^/N m for the uncoated steel. To demonstrate the adaptability of the coatings to the different hydrocarbon-rich environments, the Co5NiP was further tested in an ethanol vapor and nitrogen gas mixture. The results indicated a low and stable coefficient of friction and minimal wear of the surfaces. The origin of such an improvement has been attributed to the formation of protective carbon-based films inside the wear tracks, as suggested by the elemental mapping analysis. The Raman spectroscopy results revealed the amorphous carbon nature of such films. These carbon films assist the coatings in improving friction and wear performance during sliding.

## Data Availability

The authors confirm that the data supporting the findings of this study are available within the article.
